# Editorial: Risk assessment of mycotoxins in food

**DOI:** 10.3389/fnut.2023.1145998

**Published:** 2023-02-20

**Authors:** Nada El Darra, Nabil Grimi, Ian A. Watson, André El Khoury

**Affiliations:** ^1^Faculty of Heath Sciences, Beirut Arab University, Beirut, Lebanon; ^2^Université de Technologie de Compiègne, ESCOM, TIMR (Integrated Transformations of Renewable Matter), Centre de Recherche Royallieu, Compiègne, France; ^3^Systems, Power and Energy Research Division, School of Engineering, University of Glasgow, Glasgow, United Kingdom; ^4^Unité de Recherche Technologies et Valorisation Agro-Alimentaire (UR-TVA), Centre d'Analyses et de Recherche (CAR), Faculty of Sciences, Saint-Joseph University of Beirut, Beirut, Lebanon

**Keywords:** risk, mycotoxins, food, feed, contaminants

Mycotoxins are natural food and feed contaminants produced as secondary metabolites of filamentous fungi with known toxic and carcinogenic effects.

Food crops are prone to fungal contamination in the pre-harvest and at storage, especially during poor agricultural and storage practices (1). Therefore, the risk of contamination with mycotoxins can occur during cultivation, processing, transportation, or storage (2).

Climate conditions can provide ideal conditions for the growth and proliferation of fungi as well as mycotoxin production (3). The warmer the climate, the greater the chance of contamination by these toxic metabolites (4). Note that, beside high temperatures (>25°C), an increased relative humidity (RH) (>60%) can also play a significant role in the fungal infestation and the occurrence of different mycotoxins (5). It is worth also to mention that climate change-related abiotic factors (especially increased temperature, elevated CO_2_ and extremes in water availability) present an impact on the relative risks of mycotoxin contamination and its consequence on food safety and security. In this respect, the last report of the Intergovernmental Panel on Climate Change (CC) revealed that warming of the climate system could impact the resilience of different toxigenic species and their ability to produce mycotoxins (6).

The exposure of humans to mycotoxins is a life-threatening problem, especially due to their natural co-occurrence, which could exhibit greater toxicity and carcinogenicity than exposure to a single mycotoxin. Ingestion of high levels of certain mycotoxins, such as aflatoxins, can cause acute toxicity, leading to a serious illness or even death. Long-term exposure to high levels of these metabolites can also cause more serious health problems such as cancer, immune system suppression, and damage to the liver and kidneys (7, 8).

The European Union (EU) has a rapid alert system for food and feed (RASFF) in place to ensure that food and feed products imported into the EU are safe for consumption. The RASFF is designed to allow for rapid communication and coordination among EU member states and other countries in the event of a food safety incident and it is used to identify and respond to potential risks to human and animal health, and to prevent unsafe products from entering the EU market.

The RASFF annual report for 2016 indicates that mycotoxins were the top hazard for border rejection notifications in the European Union, and, according to the annual report for 2019, mycotoxins were still considered among the top issues for products from non-member countries, with 400 notifications (9). This highlights the ongoing concerns around mycotoxins in food products and the importance of continued monitoring and testing to ensure that they are safe for consumption.

Naturally occurring food/feed contaminants have become a significant global issue due to animal and human health implications. Acute and chronic exposure to mycotoxins can lead to teratogenic, mutagenic, carcinogenic, nephrotoxic, hepatotoxic, and immunotoxic effects on different organs. To protect the consumer from the harmful effects of these compounds, the European Union has established Maximum Levels (MLs) for mycotoxins in foods and feeds. The EU-legislation comprises several legally binding regulations. The enforceable MLs for aflatoxins (AFs), ochratoxin A (OTA), deoxynivalenol (DON), fumonisins (FBs), and zearalenone (ZEN) in various foods are specified in the Commission Regulation (EC) No 1881/2006 (10). Therefore, we aimed in this Research Topic to emphasize three major areas of on-going research on mycotoxins, starting by emphasizing on the importance of considering animal feed as a source for food contamination, assessing aflatoxin production in herbs originating from different countries and understanding the mechanism resulting from the circulation of mycotoxins in the body and their effect on the various tissues.

Ceniti et al. emphasized the importance of considering contaminated animal feed, with fungi or related mycotoxins, as a serious constraint to animal and human health and productivity. In the dairy sector, consumption of contaminated hay with fungi can cause mycotoxicoses in animals with economic losses in the dairy cow production system. This research has screened the viable molds (filamentous microfungi) and mycotoxins isolated from different lots of hay (*n* = 55) collected from 20 dairy farms located in South Italy. Many fungal species were identified as *Cladosporium cladosporioides* (*n* = 46, 84%), the major contaminant. *Aspergillus flavus* related to aflatoxin contamination was isolated in (*n* = 11, 20%) of the samples. All the hay samples were found to be scarcely contaminated with AFB1, with values from 0.0020 to 0.0077 mg/kg, below the acceptable limits (0.02 mg/kg) set by European Union (EU legislation). This study reported a significant increase presence of AFB1 for hay samples presenting a moisture between 15 and 19%. Therefore, a thorough monitoring of AFB1 in hay should be conducted, especially for the feed presenting high moisture and crude ash content, to prevent to reduce the exposure of humans and animals to mycotoxins.

Victor Jeyaraj et al. reported the occurrence of aflatoxin contamination herbal products. Aflatoxin are mycotoxins produced by different fungal species especially *Aspergillus Parasiticus* and *Aspergillus Flavus*. The international Agency for Research on Cancer (IARC) has classified aflatoxins as Group I carcinogens. The contamination of herbs with afltoxins could be mainly due to the drying process, laying down on the ground in the open air where the climatic conditions are ideal for growth of molds and production of mycotoxins. This review has screened research work assessing aflatoxins occurrence in herbs originating from different countries Malaysia, Indonesia, Kenya, Brazil, Nigeria, Thailand, South Africa, and Morocco. The significant occurrence of alfatoxins in herbs coming from the different countries emphasize the significance that should be given to contamination of aflatoxin in herbal products. It is to be noted that the percentages of aflatoxin contamination were the highest in South africa (*n* = 16, 94%), followed by Kenya (*n* = 100, 69%), Morocco (*n* = 129, 59%), Brazil (*n* = 91, 54.9%), Malaysia, Indonesia (*n* = 23, 20%), Nigeria (*n* = 210, 18.6%), and the lowest percentage for Thailand (*n* = 28, 18%). This study has reported high-performance liquid chromatography coupled with mass spectrometry as the best reliable method to determining aflatoxin levels in herbal products. However, this method is not suitable for farmers. Thus, the importance of developing tests to determine and quantify aflatoxin in herbal products is of high interest.

Righetti et al. have screened red yeast rice (RYR) food supplements for their mycotoxin content. The samples of RYR (*n* = 37), were bought mainly on internet web sites (24) but also from local pharmacies in Italy (13) between September 2020 and February 2021. The mycotoxin assesment reported the presence of citrinin (CIT) in all the products with a range from 100 to 2,5100 μg/kg, out of which only four products were compliant with maximum EU levels for CIT. It is to be noted that four contaminated products were labeled as “citrinin free.” This study has emphasized the importance of implementing a more rigorous standardization and monitoring for mycotoxin content to extrapolate data for risk assessment with respect to the prevalence of citrinin in food supplements.

Malvandi et al. have reported that mycotoxins could affect different cells throughout their circulation. Mycotoxins present an oxidative nature that could also be enhanced by subchronic co-exposure to mycotoxins. They generate free radicals, reducing antioxidant protection, triggering lipid peroxidation and damaging some biological molecules, resulting in mycotoxins-assisted oxidative imbalance leading to an oxidative stress, apoptosis and other cytotoxic effects of mycotoxins. Mycotoxins get incorporated into membrane structures causing various detrimental changes. They could be a source of low-level toxicity in the bone marrow microenvironment and arterial dysfunction. They could also alter the function of cardiomyocytes with a risk of heart injury. The co-occurrence of mycotoxins can promote the dysfunction of osteoblast with an increased risk of bone fracture. Malvandi et al. presents a novel understanding of complex mechanisms resulting from co-exposure to mycotoxins and their subsequent effect in circulation.

In conclusion, the research on this topic highlights the importance of screening fungi and mycotoxins in animal food and feed. Mycotoxins can be a significant concern for feed safety, as they can cause a variety of health problems in animals if ingested. Contaminated animal feed can lead to decreased animal performance, reduced productivity, and even death. In addition, mycotoxins can also end up in animal-derived products, such as milk, dairies and meat, which can lead to the exposure of humans to these products.

Herbs can be susceptible to mycotoxin contamination, just like any other food crop, and can cause gastrointestinal and adverse central nervous symptoms in humans if contaminated. Thus, the risk of their contamination with mycotoxins should not be underestimated and the importance of assessing the risk of their consumption on a daily basis is essential to guarantee their safety.

In the end, a focus on mycotoxins and their potential health effects represent an opportunity to strengthen our knowledge in understanding their mechanistic in biological systems and would help to identify new strategies for preventing and mitigating their impact, as well as to develop new strategies for preventing and mitigating their impact.

## Author contributions

NE and AE have made substantial, direct, and intellectual contribution to the work and approved it for publication. NG and IW have reviewed the paper. All authors contributed to the article and approved the submitted version.

## References

[1] Piotrowska M Slizewska K Biernasiak J. Mycotoxins in cereal and soybean-based food and feed. In: Brazil: Soybean-Pest Resistance. (2013). p. 185–230. 10.5772/54470

[2] Macri AM Pop I Simeanu D Toma D Sandu I Pavel LL Mintas OS. The occurrence of aflatoxins in nuts and dry nuts packed in four different plastic packaging from the romanian market. Microorganisms. (2021) 9:61. 10.3390/microorganisms9010061 33379317 PMC7823895

[3] Schweiggert U Carle R Schieber A. Conventional and alternative processes for spice production–a review. Trends Food Sci Technol. (2007) 18:260–8. 10.1016/j.tifs.2007.01.005

[4] Cüce M. Incidence of aflatoxins, ochratoxin A, zearalenone, and deoxynivalenol in food commodities from Turkey. J Food Saf. (2020) 40:e12849. 10.1111/jfs.12849

[5] Thanushree MP Sailendri D Yoha KS Moses JA Anandharamakrishnan C. Mycotoxin contamination in food: an exposition on spices. Trends Food Sci Technol. (2019) 93:69–80. 10.1016/j.tifs.2019.08.010

[6] Perrone G Ferrara M Medina A Pascale M Magan N. Toxigenic fungi and mycotoxins in a climate change scenario: ecology, genomics, distribution, prediction and prevention of the risk. Microorganisms. (2020) 8:1496. 10.3390/microorganisms8101496 33003323 PMC7601308

[7] Al Jabir M Barcaru A Latiff A Jaganjac M Ramadan G Horvatovich P. Dietary exposure of the Qatari population to food mycotoxins and reflections on the regulation limits. Toxicol Rep. (2019) 6:975–82. 10.1016/j.toxrep.2019.09.009 31673499 PMC6816139

[8] Coppock RW Christian RG Jacobsen BJ. Aflatoxins. In: Veterinary Toxicology. Elsevier (2018). p. 983–94. 10.1016/B978-0-12-811410-0.00069-6

[9] RASFF. Rapid Alert System for Food and Feed Annual Report 2020. (2020). Available online at: https://Food.Ec.Europa.Eu/System/Files/2021-08/Rasff_pub_annual-Report_2020.Pdf.

[10] Union E. Commission Regulation (EC) No 1881/2006 of 19 December 2006 setting maximum levels for certain contaminants in foodstuffs. Off J Eur Union. (2006) 49:5–24.

